# The dual effects of macrophage-derived extracellular vesicles on tumor cell behavior: mechanisms and clinical potential

**DOI:** 10.3389/fonc.2025.1586083

**Published:** 2025-05-23

**Authors:** Jia-Wen Tian, Yu-Han Fang, He-Jing Zhang, Zi-Li Yu

**Affiliations:** ^1^ State Key Laboratory of Oral & Maxillofacial Reconstruction and Regeneration, Key Laboratory of Oral Biomedicine Ministry of Education, Hubei Key Laboratory of Stomatology, School & Hospital of Stomatology, Wuhan University, Wuhan, China; ^2^ College of Life Sciences, Wuhan University, Wuhan, China; ^3^ Center for Oral and Maxillofacial Surgery at Optics Valley Branch, School & Hospital of Stomatology, Wuhan University, Wuhan, China; ^4^ Department of Oral and Maxillofacial Surgery, School and Hospital of Stomatology, Wuhan University, Wuhan, China

**Keywords:** extracellular vesicles, macrophage, tumor cell, intercellular communication, clinical potential

## Abstract

Macrophages, key players in the immune system, exhibit diverse roles in tumor progression and regulation. Macrophages release extracellular vesicles (EVs), membrane-bound particles that facilitate intercellular communication and cargo transfer. Macrophage-derived EVs (M-EVs) demonstrate a complex dual function in tumor development, with their effects dependent on their origin and the tumor microenvironment. M1-EVs show anti-tumor properties by reversing immune escape, while M2-EVs promote tumor biogenesis, invasion, metastasis, and therapeutic resistance. Tumor-associated macrophage-derived EVs (TAM-EVs) generally facilitate tumor progression but may exhibit anti-tumor characteristics in specific cancers. M-EVs, particularly M1-EVs, show promise as drug delivery vehicles in tumor-targeted therapy due to their targeting capabilities and ability to cross physiological barriers. Despite challenges in clinical application, ongoing research aims to harness the potential of M-EVs for more effective and personalized cancer treatments. This review summarizes how M-EVs influence tumor cell behavior, their mechanisms of action, and the challenges related to specificity, isolation, and clinical application. Collectively, this comprehensive analysis not only provides researchers with a better understanding of the complex roles of M-EVs in cancer biology but also lights the way for innovative therapeutic strategies, potentially advancing the development of more effective and personalized cancer treatments.

## 1 Introduction

Macrophages, as integral components of the human immune system, play a vital role in the body’s defense mechanism against various pathogens and diseases (1). Macrophages are abundant and diverse, adapting to different physiological contexts to maintain homeostasis (2). Beyond their general immune functions, macrophages also significantly impact cancer progression. They are not merely passive bystanders but active participants, influencing tumor progression and therapeutic processes through intricate cellular communication (3). Macrophages exhibit remarkable plasticity in response to different stimuli, leading to a spectrum of activation states, most notably classified into M1 and M2 phenotypes. M1 macrophages are traditionally associated with pro-inflammatory responses and anti-tumoral effects, while M2 macrophages generally support tissue repair and tumor progression (4). Furthermore, tumor-associated macrophages (TAMs), which represent a unique and significant population within the tumor microenvironment (TME), contribute to the complexity of the tumor landscape (5).

Macrophages influence tumor progression primarily through intercellular communication, a key aspect of regulating tumor behavior (6). Extracellular vesicles (EVs) are small, membrane-bound particles released by cells into the extracellular environment. EVs, particularly those originating from macrophages (M-EVs) which include vesicles derived from various macrophage phenotypes, have emerged as significant mediators of intercellular communication (7, 8). M-EVs can carry a wide range of bioactive molecules, such as proteins, lipids, and nucleic acids, to recipient cells, affecting various processes including tumor growth, metastasis, immune modulation, and response to therapy (9, 10). Their ability to encapsulate and transport these molecules makes them critical players in the dynamic interplay within the TME (11, 12). The mechanisms through which M-EVs exert their influence on tumor cells are as diverse as they are complex. They can promote tumor growth and metastasis, contribute to the creation of an immunosuppressive TME, or, conversely, facilitate anti-tumor immune responses depending on their molecular composition and the context of their release (13, 14). This nuanced interplay highlights the potential of M-EVs both as biomarkers for cancer progression and as targets or vehicles for therapeutic intervention (15–17). Their ability to encapsulate and protect therapeutic agents, combined with their inherent targeting capabilities, opens new possibilities for precision medicine (18). However, harnessing the full therapeutic potential of M-EVs requires a deeper understanding of their biogenesis, the specificity of their targeting mechanisms, and the functional consequences of their interaction with tumor cells.

This review explores the intricate ways in which M-EVs influence tumor cell behavior, detailing their mechanisms of action and potential in cancer treatment. As we delve into the complexities of M-EVs’ roles in cancer dynamics, we confront ongoing challenges related to their specificity, isolation, and clinical application. Nevertheless, the potential of M-EVs in advancing targeted and personalized approaches in cancer therapy is notable, positioning them as an innovative strategy in oncological research.

## 2 Overview of macrophage-derived extracellular vesicles

**Figure 1:**
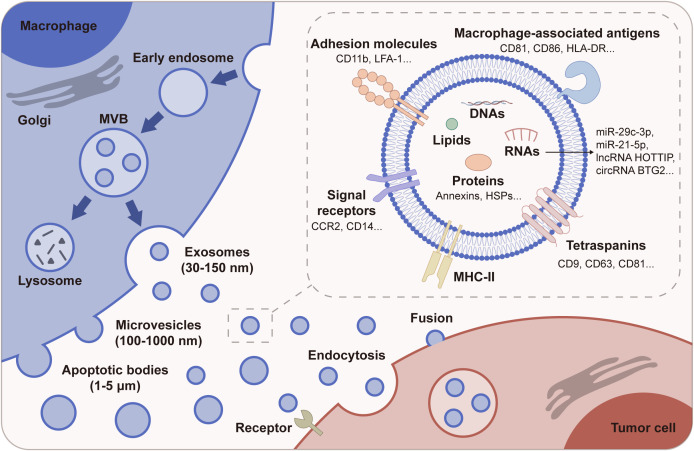
Biogenesis and composition of M-EVs. M-EVs are divided into exosomes, MiVs, and apoptotic bodies based on their biogenesis and size. Exosomes are formed within the endosomal pathway, evolving from early endosomes to MVBs, and are released when MVBs fuse with the plasma membrane. MiVs are generated by direct outward budding and fission from the plasma membrane. Apoptotic bodies arise from membrane blebbing during programmed cell death. M-EVs carry a rich cargo, including proteins such as MHC-II and tetraspanins, and other molecules like nucleic acids and lipids. M-EVs can affect tumor cell behavior through endocytosis, membrane fusion, or receptor-ligand interactions.

M-EVs represent a specialized group of vesicles emanating from macrophages, which are pivotal immune cells involved in both innate and adaptive responses. These vesicles encompass various forms including exosomes, microvesicles (MiVs), and apoptotic bodies, each distinguished by their size, biogenesis pathway, and functional implications (19, 20). The genesis of M-EVs is a tightly regulated process. Exosomes originate from the inward budding of endosomal membranes to form multivesicular bodies (MVBs), which then fuse with the plasma membrane to release these vesicles into the extracellular space. MiVs, conversely, emerge through the outward budding and fission of the plasma membrane (21) (**Figure 1**). This diversity in biogenesis directly influences their cargo and functions, reflecting the macrophage’s state and activity within the immune system.

M-EVs are rich in a variety of biomolecules, including proteins, lipids, and a spectrum of nucleic acids such as mRNA, miRNA, and other non-coding RNAs. This molecular payload is reflective of the macrophage’s functional state and can profoundly influence recipient cells. Importantly, the biological effects of M-EVs are highly context-dependent, shaped by the type of macrophages (e.g., M1 *vs*. M2), the TME, and the particular cancer type. M-EVs derived from classically activated (M1) macrophages (M1-EVs) are typically characterized by the presence of pro-inflammatory cytokines, chemokines, and enzymes like TNF-α, IL-6, and iNOS, while M-EVs originating from alternatively activated (M2) macrophages (M2-EVs) often contain anti-inflammatory molecules such as IL-10 and TGF-β (9). This polarization is influenced by the TME, where factors such as tumor-secreted cytokines, stromal cell interactions, and local metabolic conditions not only dynamically regulate macrophage activation but also impact the biogenesis and molecular composition of M-EVs (5, 22). Environmental stressors—such as hypoxia and tumor-derived EVs—can influence the secretion and functional profiles of M-EVs by reprogramming macrophage metabolism and activation states, thereby altering their EV cargo composition (23, 24). These context-dependent regulatory mechanisms modulate the molecular composition of M-EVs, endowing them with either tumor-promoting or tumor-inhibiting properties. In turn, these M-EVs influence tumor cell behavior and immune responses, highlighting their role in the dynamic crosstalk between macrophages and the TME.

**Figure 2:**
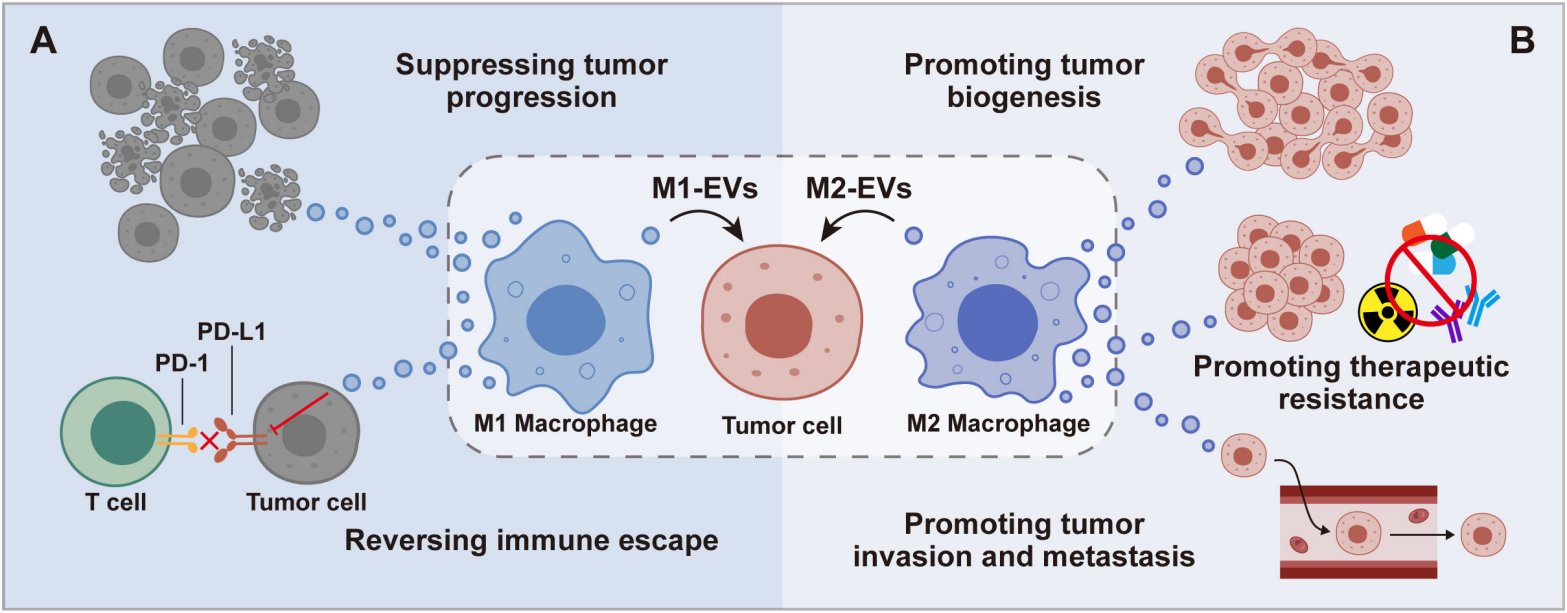
Impact of M1 and M2 Macrophage-derived EVs on Tumor Cells. M1-EVs are shown to inhibit tumor progression and combat immune suppression **(A)**, while M2-EVs facilitate tumor development, invasion, metastasis, and contribute to therapeutic resistance **(B)**, emphasizing their diverse influence on cancer dynamics.

Serving as key mediators of intercellular communication, M-EVs can profoundly modulate immune responses, influence the behavior of tumor cells, and contribute to the dynamics of the TME (25–27). Their roles are inherently dualistic: M1-EVs often exhibit anti-tumor properties, while M2-EVs may support tumor growth and metastasis by fostering angiogenesis, suppressing immune responses, and enhancing tumor cell survival and proliferation (28–30) (**Figure 2**). This dual functionality underscores the potential of M-EVs in cancer research. M-EVs’ ability to carry signals that influence tumor behavior shows promise for developing new cancer treatments. By understanding how M-EVs work, scientists hope to use them for targeted therapy, offering personalized approaches to cancer treatment.

## 3 Impact of M1 macrophage-derived EVs on tumor cells

### 3.1 M1-EVs suppress tumor progression

**Figure 3:**
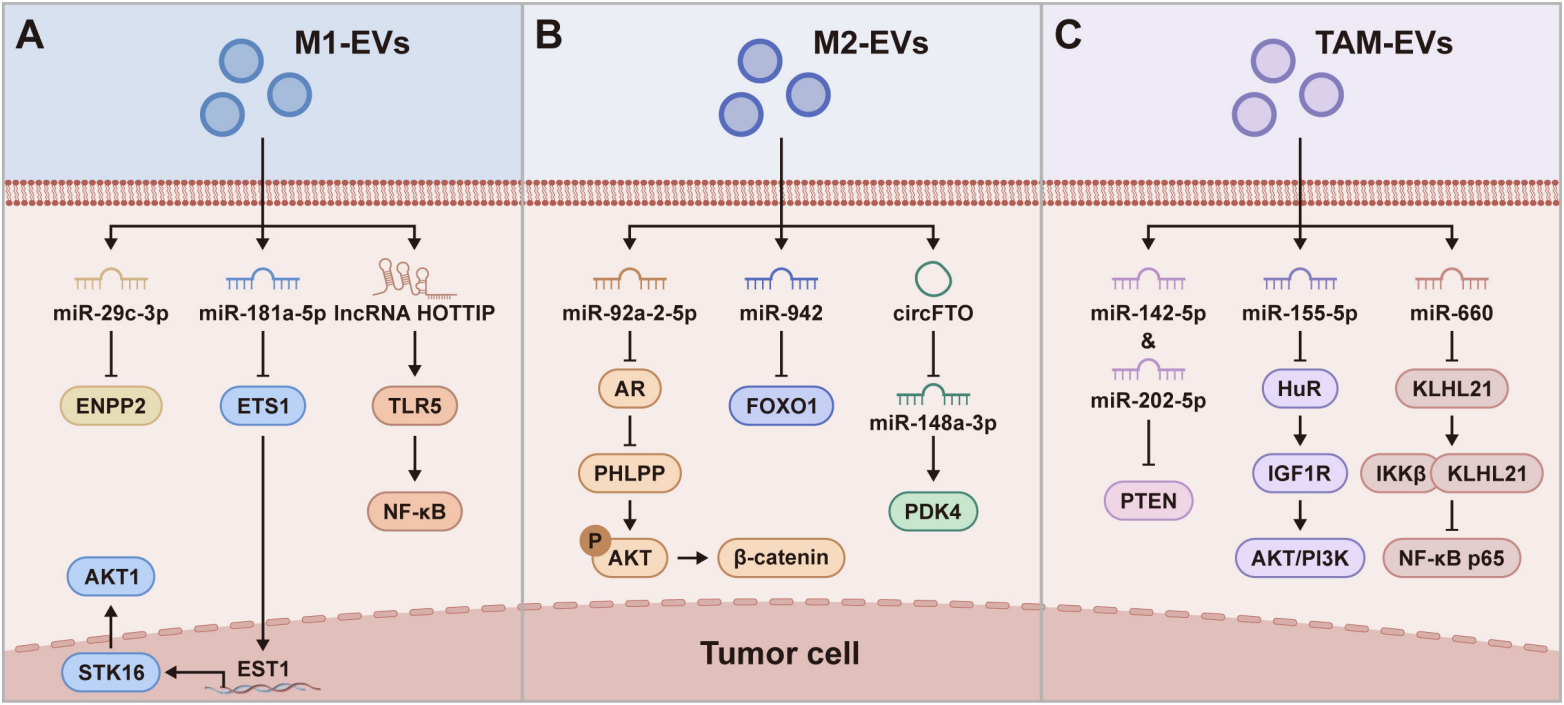
Molecular mechanisms on which M1-EVs, M2-EVs, and TAM-EVs regulate the tumor cells. **(A)** M1-EVs regulate tumor cells via pathways such as miR-29c-3p/ENPP2, miR-181a-5p/ETS1/STK16, and lncRNA HOTTIP/TLR5/NF-κB, leading to suppression of tumor cell migration and invasion, and induction of tumor cell apoptosis. **(B)** M2-EVs regulate tumor cells via pathways including miR-92a-2-5p/AR/PHLPP/AKT/β-catenin, miR-942/FOXO1, and circFTO/miR-148a-3p/PDK4, thereby promoting tumor cell invasion, metastasis, and glycolysis. **(C)** TAM-EVs regulate tumor cells via pathways such as miR-142-5p/PTEN, miR-155-5p/HuR/IGF1R, and miR-660/KLHL21/IKKβ/NF-κB, promoting tumor progression.

M1-EVs play a pivotal role in combating tumor progression by suppressing the proliferation, migration and invasion of tumor cells, as demonstrated in numerous studies (**Table 1**, **Figure 3A**). These findings highlight the specific anti-tumoral properties of M1-EVs, which distinguish them from other macrophage-derived vesicles.

**Table 1:** M-EVs regulate tumor cells through multiple mechanisms.

Cargo	Donor cell	Tumor cell line	Molecular mechanism	Biological function	Ref.
miR-29c-3p	M1 macrophage	Melanoma	ENPP2	Alleviating tumor cell migration and invasion	(31)
miR-181a-5p	M1 macrophage	LUAD	miR-181a-5p/ETS1/STK16	Inhibiting tumor cell viability and promoting tumor cell apoptosis	(35)
lncRNA HOTTIP	M1 macrophage	HNSCC	Activating TLR5/NF-κB	Suppressing tumor progression	(14)
/	M1 macrophage	BC	Activating NF-кB	Enhancing tumor cell cycling	(34)
miR-21	M2 macrophage	GBM	Downregulating PEG3	Promoting tumor cell proliferation, migration and invasion	(25)
miR-21-5p	M2 macrophage	RCC	PTEN/Akt	Promoting tumor migration and invasion	(36)
miR-27a-3p	M2 macrophage	HCC	Downregulating TXNIP	Promoting tumor cell stemness, proliferation, drug resistance, migration, invasion and *in vivo* tumorigenicity	(37)
miR-27a-3p, miR-22-3p and miR-221-3p	M2 Macrophage	GBM	CHD7/RelB/P50 and CHD7/p-STAT3	Inducing proneural-to-mesenchymal transition and promoting radioresistance	(38)
miR-92a-2-5p	M2 Macrophage	HCC	AR/PHLPP/p-AKT/β-catenin	Promoting tumor cell invasion	(39)
miR-155-5p and miR-21-5p	M2 macrophage	CC	Downregulating BRG1	Promoting tumor cell migration and invasion	(40)
miR-186-5p	M2 macrophage	CC	Inhibiting DLC1	Promoting tumor cell proliferation and motility	(41)
miR-501-3p	M2 macrophage	LC	Downregulating WDR82	Promoting tumor cell growth	(42)
miR-501-3p	M2 macrophage	PDAC	Downregulating TGFBR3	Promoting tumor formation and metastasis	(26)
miR-942	M2 macrophage	LUAD	Downregulating FOXO1	Promoting tumor metastasis	(13)
Circ_0020256	M2 macrophage	CCA	miR-432-5p/E2F3	Promoting tumor cell proliferation, migration, and invasion	(43)
circFTO	M2 macrophage	NSCLC	miR-148a-3p/PDK4	Promoting tumor cell progression and glycolysis	(44)
lncRNA AGAP2-AS1	M2 macrophage	Radioresistant NSCLC	miR-296/NOTCH2	Strengthening tumor cell radioresistance	(45)
ApoE	M2 macrophage	Gastric cancer	PI3K-Akt	Promoting tumor cell migration	(46)
miR-125a/b	TAM	HCC	Targeting CD90	Suppressing tumor cell proliferation, stem cell properties and migration	(27)
miR-142-5p, miR-202-5p	TAM	PDAC	Suppressing PTEN	Promoting tumor cell invasiveness and migratory potential	(47)
miR-155-5p	TAM	786-0	Enhancing HuR-mediated mRNA stability of IGF1R	Promoting tumor progression	(23)
miR-660	TAM	Breast cancer tissue	Blocking KLHL21/IKKβ axis	Promoting tumor metastasis	(48)
Arginase-1	TAM	GBM	/	Promoting tumor cell proliferation and migration	(49)
lncRNA LIFR-AS1	Macrophage	OS	miR-29/NFIA	Promoting tumor growth and metastasis	(50)
GARS1	Macrophage	H460	/	Inhibiting tumor cell growth	(51)
ADAM15	Macrophage	Ovarian cancer	/	Suppressing tumor cell growth and migration	(52)
PTEN	Apoptotic tumor cell-induced macrophage	344SQ	Downregulating Akt/p38	Suppressing tumor cell invasion	(53)
IL-6	Apoptotic tumor cell-induced macrophage	BC	Increasing the phosphorylation of STAT3	Promoting tumor cell proliferation, invasion, and metastasis	(54)
circRNA BTG2	RBP-J-overexpressed macrophage	Glioma	circBTG2/miR-25-3p/PTEN	Inhibiting tumor growth	(55)

LUAD, Lung Adenocarcinoma; HNSCC, Head and Neck Squamous Cell Carcinoma; BC, Breast Cancer; GBM, Glioblastoma; RCC, Renal Cell Carcinoma; HCC, Hepatocellular Carcinoma; CC, Colon Cancer; LC, Lung Cancer; PDAC, Pancreatic Ductal Adenocarcinoma; CCA, Cholangiocarcinoma; NSCLC, Non-Small Cell Lung Cancer; OS, Osteosarcoma.

For example, M1 macrophages secrete exosomal miR-29c-3p, which targets ENPP2 in melanoma cells, reducing their migration and invasion by modulating cholesterol metabolism and extracellular matrix remodeling (31). Additionally, M1-EVs have been found to reduce cell migration in breast cancer cells by delivering miRNA-326, which downregulates NF-κB expression (32, 33). This is particularly noteworthy because NF-κB plays a central role in promoting cancer cell proliferation and survival.

However, the effects of M1-EVs on tumor cells are complex and multifaceted. For instance, while they can inhibit the migration of certain breast cancer cells, M1-EVs have also been observed to enhance the migration of breast cancer stem cells and influence the mesenchymal-epithelial transition (MET)/epithelial-mesenchymal transition (EMT) program (34). This duality in their function underscores the need for a more nuanced understanding of their role in cancer biology. Furthermore, these findings suggest that the therapeutic application of M1-EVs must be tailored to specific cancer types and stages to maximize their efficacy while minimizing unintended consequences.

### 3.2 M1-EVs reverse tumor immune escape

M1-EVs are increasingly recognized for their critical role in counteracting tumor immune escape, a process they facilitate by reducing PD-L1 expression on tumor cells. Specifically, these vesicles carry miR-16-5p, an antioncogenic factor identified for its ability to inhibit GC cell proliferation and migration by targeting SALL4 (56). Research by Li et al. has highlighted the mechanism by which M1-EVs, through miR-16-5p, suppress PD-L1 expression in GC cells, thereby diminishing the cancer’s ability to evade immune detection. This action enables T cells to more effectively recognize and combat GC cells. The interruption of PD-1/PD-L1 interactions by M1-EVs leads to enhanced T cell activation and corresponding inhibition of GC cell proliferation (57).

M1-EVs have been reported to alter the state of dormant breast cancer cells within the bone marrow. These vesicles effectively downregulate PD-L1 on tumor cells, transforming them from a dormant to an active, proliferating state and increasing their responsiveness to chemotherapy, like carboplatin (34). This finding is vital, considering bone marrow dormancy often leads to delayed breast cancer recurrence. While current evidence supports this effect, further research is needed to establish its broader significance and elucidate the underlying mechanisms. The study of M1-EVs opens new insights into their dual role in cancer therapy: while they can activate the immune response against tumors, they also have the potential to unexpectedly revive dormant cancer cells. This highlights the complex, two-fold impact of M1-EVs in cancer progression and treatment, underscoring the need for careful consideration in their therapeutic use.

## 4 Impact of M2 macrophage-derived EVs on tumor cells

### 4.1 M2-EVs promote tumor biogenesis

M2-EVs play a significant role in the initiation and progression of various cancers, primarily through the transfer of specific microRNAs that regulate key oncogenic pathways. In medulloblastoma, M2-EVs encapsulate and deliver miR-155-3p to DAOY cells, a medulloblastoma cell line. This microRNA is responsible not only for promoting the progression of these cancer cells but also for accelerating tumorigenesis *in vivo*, indicating the profound impact of M2-EVs on tumor cell behavior and disease progression (58). The influence of M2-EVs extends to meningioma as well, where they have been implicated in promoting tumor development. This effect is modifiable; blocking TGF-β signaling can partially reverse the tumorigenic influence of M2-EVs on meningioma cells (59). This suggests a potential therapeutic target in managing meningioma.

### 4.2 M2-EVs promote tumor invasion and metastasis

M2-EVs have been recognized for their complex roles in promoting tumor invasion and metastasis in various cancers, a phenomenon largely mediated by specific microRNAs and long non-coding RNAs (**Table 1**, **Figure 3B**). In lung adenocarcinoma, the role of M2-EVs is highlighted as they facilitate cell invasion and migration. This is achieved through the delivery of miR-942, which suppresses FOXO1 expression and activates the Wnt/β-catenin signaling pathway, thereby enhancing angiogenesis, a key factor in tumor growth and spread (13). The impact of M2-EVs extends to esophageal cancer as well, where they transfer lncRNA AFAP1-AS1 to the cancer cells. This transfer leads to the downregulation of miR-26a and upregulation of ATF2, thereby promoting both invasion and metastasis of esophageal cancer cells (60).

Interestingly, M2-EVs, through specific molecules such as miR-15a and miR-92a, demonstrate the potential to hinder glioma cell migration and invasion by targeting the PI3K/AKT/mTOR signaling pathway. This underscores the diverse and multifaceted functions of M2-EVs in cancer biology, as most studies report their role in enhancing tumor invasion and metastasis, while they may also have suppressive effects on tumor progression, likely depending on the specific molecular cargo they carry and the cancer type involved (61).

### 4.3 M2-EVs promote tumor therapeutic resistance

Chemotherapy, radiotherapy, and targeted therapy are key components among cancer treatments, each playing an indispensable role in the battle against this complex disease (62). Chemotherapy harnesses cytotoxic drugs to obliterate rapidly dividing cells, radiotherapy employs high-energy radiation to damage the DNA of cancer cells, and targeted therapy specifically aims at unique molecular targets associated with cancer growth (63, 64). Despite the effectiveness of these therapies, resistance development remains a significant clinical hurdle, with M2-EVs being identified as one of the contributors to this phenomenon. These vesicles transport bioactive molecules that can alter tumor cell sensitivity, leading to a subset of cancer cells that can withstand these conventional therapeutic attacks, thus presenting an obstacle to successful cancer eradication (38, 45, 65–69).

In chemotherapy resistance, pancreatic cancer cells internalize M2-EVs carrying miR-222-3p, which enhances resistance to gemcitabine by inhibiting TSC1 expression and activating the PI3K/AKT/mTOR pathway. M2-EVs not only reduce apoptosis in these cancer cells but also promote their proliferation, thereby complicating the outcomes of treatment (65). Similarly, in gastric cancer, M2-EVs containing circ 0008253 augment resistance to oxaliplatin, leading to decreased apoptosis and increased tumor cell viability (66). Moreover, gastric cancer cells exposed to M2-EVs rich in miR-21 show enhanced resistance to cisplatin, a phenomenon linked to the suppression of PTEN and activation of the PI3K/AKT pathway (67).

Radiotherapy resistance is also potentiated by M2-EVs, as they facilitate the proneural-to-mesenchymal transition in glioma stem cells via miRNAs like miR-27a-3p, exacerbating resistance and reducing the efficacy of radiotherapy (38). Additionally, M2-EVs carry lncRNA AGAP2 antisense RNA 1 (AGAP2-AS1), which strengthens radioresistance in lung cancer cells, presenting a significant barrier to successful treatment (45).

Resistance to targeted therapies, especially in non-small cell lung cancer (NSCLC) treated with EGFR-TKIs, poses a significant challenge. Yuan et al. found that extracellular vesicles from M2-EVs contribute to this resistance by affecting pathways like AKT, ERK1/2, and STAT3. This insight opens new research directions for alternative treatments in NSCLC after EGFR-TKI resistance develops (68).

M2-EVs’ role in therapeutic resistance underscores a critical area for further investigation in cancer treatment, potentially leading to enhanced strategies for managing resistance.

## 5 Impact of tumor-associated macrophage-derived EVs on tumor cells

TAMs, key cellular components of the tumor microenvironment, play a critical role in cancer progression. They diverge from the binary M1/M2 macrophage classification, instead existing in a continuum that spans these two states and adapts to the tumor’s dynamic environment (70). TAMs’ functions are intricately linked to the type and stage of cancer and the specificities of the microenvironment. Often, they exhibit an M2-like phenotype, suppressing immune responses and aiding in tumor growth (71).

TAM-EVs, sourced from macrophages isolated directly from tumor tissues, offer a more accurate representation of the TME than those from cultured macrophages. This enhanced representation provides a more precise reflection of the complex interactions within the TME, which is essential for comprehending tumor progression and developing targeted therapeutic strategies. Proteomic analyses have identified classic EV markers (ALIX, CD63, TSG101, CD81, CD9) alongside macrophage-specific markers such as MRC1/CD206, confirming the macrophage origin). Notably, TAM-EVs exhibit a Th1/M1 polarization signature, carrying inflammatory mediators and immune modulators, despite their parent TAMs often displaying an M2-like phenotype (9). Beyond proteins, TAM-EVs carry bioactive lipids (cholesterol, sphingolipids, glycosphingolipids) and signaling molecules (ceramides, sphingosine-1-phosphate) involved in immune regulation. They also transport miR-511-3p, a macrophage-specific miRNA that modulates immune responses and inflammation. These components suggest that TAM-EVs may regulate inflammation and immune responses, influencing tumor progression (9).

TAM-EVs have a substantial regulatory impact on tumor cells (**Table 1**, **Figure 3C**). Emerging evidence indicates that the functions of TAM-EVs vary across different cancer types, reflecting their complex roles in tumor progression. In cancers such as hepatocellular carcinoma (HCC), breast cancer, and pancreatic ductal adenocarcinoma, TAM-EVs primarily facilitate tumor growth, promoting proliferation, metastasis, and immune evasion. In contrast, in colorectal cancer, TAM-EVs exhibit characteristics akin to anti-tumor M1 macrophages. In HCC, TAM-EVs significantly enhance tumor cell growth and the properties of cancer stem cells. These EVs, characterized by low levels of miR-125a and miR-125b, have been shown to promote HCC cell proliferation, sphere formation, and metastasis. This effect is mediated through the modulation of CD90, a critical stem cell marker in HCC, demonstrating how TAM-EVs can alter cancer cell phenotypes (27). TAM-EVs significantly impact breast cancer by disrupting tumor-suppressive mechanisms. Laden with miR-660, they impede the tumor suppressor KLHL21, activating the NF-κB p65 pathway, which is key to enhancing breast cancer cell invasion and migration. This disruption, primarily through the suppression of KLHL21 by miR-660 in TAM-EVs, not only accelerates breast cancer progression but also greatly increases the risk of lymph node metastasis (48). In pancreatic ductal adenocarcinoma, the transfer of miR-202-5p and miR-142-5p by TAM-derived exosomes targets the PTEN gene. This suppressive activity on PTEN promotes the invasiveness and migratory potential of pancreatic ductal adenocarcinoma cells, fostering metastasis (47).

Interestingly, research on colorectal cancer has unveiled that TAM-EVs exhibit characteristics akin to the anti-tumor M1 macrophage phenotype, contrary to the typical immunosuppressive behavior of their originating TAMs ​​​​ (9, 72). Also, proteins found in TAM-EVs have been associated with a favorable prognosis in cancer patients, whereas proteins specifically expressed in the source TAMs show no clear correlation with clinical outcomes (9). This discrepancy may be attributed to the selective packaging of EV cargo, wherein TAMs actively sort specific proteins into EVs rather than passively releasing cellular content (73). Moreover, research suggests TAM-EVs could influence lipid metabolism in cancer cells, shifting from a COX2/PGES pathway that promotes tumor growth to a COX1/TBXAS1 pathway, potentially curtailing the tumor-supporting effects of certain prostaglandins (9). This revelation underscores the nuanced role of TAM-derived vesicles in oncology, positioning them as potential therapeutic agents that could activate anti-tumor immune responses despite the generally immunosuppressive milieu created by TAMs.

Given the phenotypic and functional heterogeneity of TAMs across tumor types and stages, the composition and effects of TAM-EVs are likewise diverse. This variability adds complexity to the understanding of their roles in tumor biology and poses challenges for their standardized therapeutic application. Further characterization of TAM subsets and their EV profiles in specific tumor contexts is essential to harness their full clinical potential.

## 6 Clinical potential of tumor-targeted M-EVs

EVs have emerged as a promising vehicle for drug delivery, thanks to their unique capability to encapsulate a wide array of therapeutic agents (74, 75). Their ability to encapsulate a wide range of therapeutic agents, along with inherent targeting capabilities mediated by specific surface markers, enables precise delivery to particular cell types or tissues and enhances therapeutic efficacy (18). Compared to cellular counterparts, EVs are more advantageous in terms of storage and safety, offering easier preservation and a lower risk of adverse immune reactions (76). Relative to synthetic systems such as lipid nanoparticles, EVs demonstrate superior biocompatibility, reduce immunogenicity, and decrease reliance on chemical modifications for targeted delivery (77, 78). These attributes collectively underscore the clinical potential of EVs, including M-EVs, as a biologically derived and translationally favorable platform for targeted drug delivery, especially in tumor-specific therapeutic applications. While certain technical aspects, such as drug loading efficiency and scalability, remain to be refined, the tumor-homing capacity and overall biological compatibility of EVs continue to make them a highly promising candidate for further development.

Building upon these advantages, M-EVs have garnered significant attention in cancer research due to their tumor-targeting capabilities and potential for therapeutic delivery. The ability of M-EVs to cross physiological barriers, like the blood-brain barrier (BBB), extends their applicability to challenging treatment areas. M1-EVs, in particular, are highlighted for their anti-tumor properties, making them ideal carriers for chemotherapy agents. The ongoing research and application of tumor-targeting M-EVs for drug delivery positions them as key players in the development of new cancer therapies, opening the door to more precise, targeted, and individualized treatment options.

**Figure 4:**
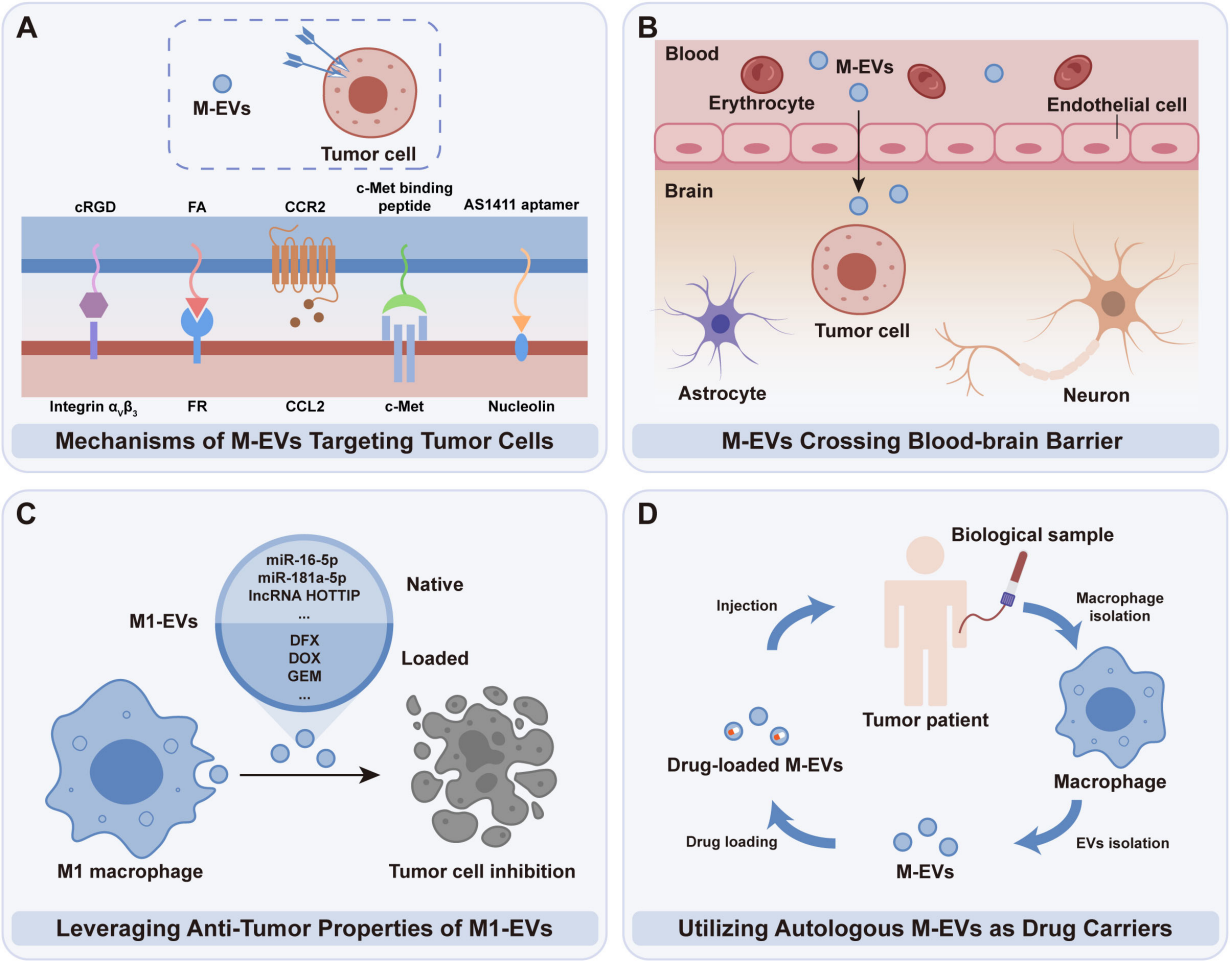
Clinical potential of tumor cell-targeted M-EVs. **(A)** M-EVs harness specific receptors overexpressed on tumor cells to deliver therapeutic agents selectively. **(B)** M-EVs’ unique capability to penetrate the BBB positions them advantageously for delivering drugs to tumor regions. **(C)** M1-EVs, with intrinsic anti-tumor properties, are excellently suited for drug delivery applications. **(D)** Autologous M-EVs present a novel, patient-specific method for drug delivery in cancer therapy.

The specific targeting capability of M-EVs towards tumor cells presents a critical advantage in cancer therapeutics. By leveraging tumor cells’ overexpressed receptors, M-EVs deliver therapeutic agents with precision, enhancing efficacy and reducing collateral effects (**Figure 4A**). For instance, Li et al. demonstrated that folate (FA) modification enhances the tumor-targeting ability of M-EVs by taking advantage of the overexpression of FA receptors on tumor cells. FA-modified M-EVs, generated by co-culturing macrophages with DSPE-PEG-folate, exhibited significantly increased cellular uptake and tumor accumulation, with twice the accumulation observed in a BALB/c mouse 4T1 breast cancer model compared to unmodified EVs (79). MiVs, abundant in protein content from M1 macrophages, have demonstrated inherent tumor-targeting properties, partly due to C-C chemokine receptor type 2 (CCR2)-rich membranes, encouraging the homing of donor cells to tumor sites (80). These MiVs, contrasted with doxorubicin (DOX)-laden M1 macrophages, present a safer alternative for drug delivery, reducing risks associated with cellular therapies, such as unintended cell engraftment, proliferation, and off-target drug release (81). Notably, the CCR2 marker, a surface marker of M1 macrophages, is enriched in MiVs but is scarcely detected in other vesicle types like apoptotic bodies and exosomes. This difference may be due to EV biogenesis, as MiVs bud from the plasma membrane and retain surface markers like CCR2, whereas exosomes and apoptotic bodies originate from endosomes and cell fragmentation, respectively (80). However, further research is required to confirm this hypothesis.

Recent studies have enhanced the targeting capability of M-EVs towards tumor cells through additional modifications. For instance, a c-Met binding peptide with high affinity for c-Met overexpressed in triple-negative breast cancer, and a tumor-targeting peptide, cyclic RGD peptide (cRGD), binding to integrin α_v_β_3_ receptor on diffuse intrinsic pontine glioma (DIPG) cells, were immobilized on the surface of EV-coated nanoplatforms (82, 83). Additionally, AS1411 aptamer-modified M-EVs were used to fabricate a biodegradable nanoplatform, CSI@Ex-A, improving targeting capability towards glioblastoma (GBM) cells (84). These approaches significantly improved therapeutic efficiency, offering new strategies for targeted cancer therapy.

The intrinsic ability of M-EVs to traverse physiological barriers, notably the BBB, offers a distinct advantage in delivering therapeutic agents directly to challenging areas, including the central nervous system and tumor regions (**Figure 4B**). This minimizes unintended impacts on non-targeted tissues. The mechanism whereby M-EVs utilize integrin LFA-1 to interact with ICAM-1 on cerebral endothelial cells is instrumental in enhancing their penetrative capability across the BBB (85). This feature is particularly advantageous in brain tumor therapies. A novel nano drug delivery system has been developed to effectively target DIPG, a formidable pediatric brain tumor. The system, which embeds panobinostat and PPM1D-siRNA within specially engineered M-EVs modified with the tumor-targeting peptide cRGD, showcases improved delivery efficiency and therapeutic impact, enabling the drugs to cross the BBB and directly target DIPG cells, consequently extending the survival time of the model mice (83).

As a group of M-EVs, M1-EVs inherently possess anti-tumor capabilities, making them an optimal choice for drug delivery vehicles (**Figure 4C**). The utilization of M1-EVs for drug transport significantly bolsters chemotherapy’s effectiveness by triggering apoptotic pathways, as indicated by the elevated levels of apoptosis markers such as Bax and caspase-3 (15, 86). One possible mechanism underlying this effect is that M1-EVs can transport miR-let-7b-5p to tumor cells, where miR-let-7b-5p regulates the GNG5 protein level, leading to increased expression of the pro-apoptotic protein Bax and promoting tumor cell apoptosis (87). This action is instrumental in fostering an immune milieu that proactively inhibits tumor growth, thereby establishing M1-EVs as prime vehicles for therapeutic intervention, thanks to their inherent anti-tumor functionality (88, 89).

Building upon these therapeutic advantages, it is also important to consider the clinical source of M-EVs. For translational applications, M-EVs can be obtained from established macrophage cell lines or, preferably, from the patient’s own macrophages (**Figure 4D**). These autologous M-EVs are inherently biocompatible and less likely to trigger antigenic responses compared to allogeneic or donor-derived EVs (90, 91). Although the preparation of autologous M-EVs can be more time- and resource-intensive, ongoing technological advances are steadily improving their feasibility for timely and personalized therapeutic applications. In contrast, standardized, donor-derived EV therapies offer scalability and immediate availability, but require thorough validation to address immunological concerns and ensure consistent efficacy across patient populations (92).

Based on the current advancements, M-EVs have been utilized to deliver a variety of chemotherapeutic drugs, including DOX and so on, directly to tumor cells, inhibiting their proliferation and invasion. Moreover, M-EVs have been adapted to transport genetic materials like PPM1D-siRNA, offering a novel approach to gene therapy by specifically targeting and modulating the genetic pathways involved in tumor cell survival (summarized in **Table 2**).

**Table 2:** Tumor cell-targeted M-EVs are employed as drug delivery systems.

Carrier type	Effector molecules	Surface modification	Tumor cell line	Effects	Ref.
EVs	CAT, ICG	AS1411 aptamer	U87	HIF-1α activation Intracellular hypoxia Tumor cell viability	(84)
EVs	DOX, PpIX	FA	4T1	Tumor cell apoptosis Tumor growth	(79)
EVs	DOX	/	GL261	Tumor growth	(93)
M1-EVs	GEM, DFX	/	PANC-1	Intracellular iron amount Tumor cell proliferation Tumor cell attachment and migration Chemoresistance to GEM	(15)
M1-MiVs	DOX	/	SKOV3	Tumor cell death Tumor cell invasion Tumor cell chemosensitivity	(80)
EV-liposome hybrid vesicles	DOX	/	K7M2, 4T1	Tumor cell viability	(94)
EV membrane	DOX	c-Met binding peptide	MDA-MB-231	Tumor cell apoptosis	(82)
EV membrane	Panobinostat, PPM1D siRNA	cRGD	DIPG	Tumor cell viability Tumor growth Amount of pleomorphic tumor cells	(83)

CAT, Catalase; ICG, Indocyanine Green; DOX, Doxorubicin; PpIX, Protoporphyrin X; FA, Folate; GEM, Gemcitabine; DFX, Deferasirox; M1-MiVs, M1 Macrophage-derived Microvesicles.

## 7 Challenges and future perspectives

M-EVs hold immense promise for personalized and targeted cancer therapies, offering a novel platform for selective drug delivery. However, their clinical translation faces significant challenges, spanning from understanding their targeting mechanisms to addressing technical, regulatory, and safety hurdles. Overcoming these obstacles is crucial to unlock the full therapeutic potential of M-EVs.

One major hurdle lies in the scalability and specificity of their production. Current isolation processes, such as ultracentrifugation and size-exclusion chromatography, are not only labor-intensive and low-yielding, but also struggle to distinguish between EV subtypes with overlapping physical and biochemical characteristics, making it difficult to isolate vesicles with distinct functional or targeting properties (95). Recent advancements in microfluidic technologies hold promise for improving the yield and purity of M-EVs by enabling high-throughput isolation and precise selection of vesicle subtypes (96–99). These refined techniques could enhance the specificity and efficiency of EV harvesting, ensuring that only vesicles with tumor-targeting capabilities are isolated while minimizing contamination.

In addition to production challenges, the method of drug loading into EVs is critical. Post-isolation techniques such as electroporation have shown promise for enhancing drug-loading efficiency, but maintaining the integrity and stability of the EV membrane during and after loading remains crucial to ensure effective delivery and biological activity (100). Emerging approaches such as sonication, freeze-thaw cycles, and click chemistry-based drug conjugation may provide innovative solutions to enhance drug encapsulation while preserving EV functionality (101). In contrast to these post-isolation methods, biosynthetic loading strategies involve genetically engineering macrophages to express therapeutic molecules that are naturally incorporated into EVs during their biogenesis (55, 102). This strategy offers a biologically integrated alternative that may facilitate large-scale and stable EV production. In addition, a more commonly reported method involves incubating macrophages with small-molecule drugs, such as DOX or 5-aminolevulinic acid (5-ALA), which are then naturally loaded into M-EVs and used for tumor-targeted therapy (79, 103). These pre-isolation techniques avoid post-isolation modifications that may damage the EV membrane and help preserve EV structural and functional integrity. Moreover, the route of administration plays a significant role in the biodistribution and therapeutic efficacy of EVs. For clinical use, EVs can be infused either locally or systemically. Local injection can enhance delivery to the disease site and reduce systemic exposure, thereby minimizing off-target effects, particularly in diseases like metastatic ovarian cancer that are often confined within the peritoneal cavity (80). On the other hand, systemic administration (e.g., intravenous injection) is more broadly applicable but faces challenges such as rapid clearance by the reticuloendothelial system, necessitating further exploration of surface modifications, such as PEGylation or the incorporation of targeting ligands, to improve circulation time and tumor specificity (104).

The targeting mechanisms of M-EVs towards tumor cells represent an emerging field that requires deeper investigation (8). M-EVs could possess an innate ability to distinguish between malignant and non-malignant cells, potentially through the overexpressed surface proteins on tumor cells that facilitate specific receptor-mediated internalization (94). Such a mechanism could enable the targeted delivery of therapeutics to tumor sites while sparing healthy tissues, thereby improving treatment efficacy and reducing side effects. However, research in this area is still limited, and efforts to improve the targeting efficiency of M-EVs to tumor cells are ongoing. Comprehensive immunological and molecular studies are necessary to advance our understanding and optimize the design of M-EVs for better outcomes in cancer therapy.

Beyond technical hurdles, the clinical translation of M-EV-based therapies is impeded by regulatory and methodological challenges. Key concerns include the absence of standardized protocols for isolation, characterization, and quantification of M-EVs, which hampers reproducibility and complicates cross-study comparisons due to variability in isolation techniques (105). Furthermore, issues such as standardized manufacturing protocols, quality control, and compliance with Good Manufacturing Practices (GMP) must be addressed to ensure the safety and consistency of M-EV products (106). Additionally, the immunogenicity of M-EVs, especially those derived from allogeneic sources, add the risk of off-target effects remain key concerns. While M-EVs, particularly M1-EVs, show promise for targeted delivery, they may also interact with non-cancerous cells if not properly tailored, potentially leading to unwanted side effects or toxicity. This highlights the need for thorough preclinical evaluation to ensure their safety.

Despite their promise, M-EVs are not without limitations. Their heterogeneity in terms of size distribution, molecular composition, and functional properties, create challenges for standardization and reproducibility. Additionally, the context-dependent nature of their effects—driven by differences in macrophage polarization, tumor microenvironmental conditions, and cancer type—makes it difficult to generalize findings across different tumor types or patient populations. For example, the immunosuppressive properties of M2-EVs, while advantageous for certain inflammatory conditions, may also exacerbate tumor progression by facilitating immune evasion and fostering an immunosuppressive TME, thereby potentially limiting the effectiveness of M-EV-based therapies (107–109). Within the TME, the presence of other immune cells, soluble mediators (e.g., cytokines, chemokines), and tumor-derived factors can dynamically reshape the phenotypic and functional output of M-EVs, sometimes leading to unpredictable or even opposing biological outcomes (110). Currently, 3D organoids and microfluidic models are being employed to better mimic the TME, which may help overcome some of the limitations associated with the unpredictable behavior of M-EVs (111). Furthermore, their rapid clearance may limit therapeutic efficacy, while inefficient targeting can lead to off-target accumulation, potentially resulting in unintended biological effects (112). Although preclinical studies have demonstrated promising antitumor effects of M-EVs, their clinical translation remains limited. Challenges such as maintaining EV stability, precisely controlling release kinetics and cargo content, and overcoming physiological barriers *in vivo* must be addressed before broader clinical application can be realized. These concerns underline the importance of careful patient selection and monitoring during treatment to maximize therapeutic benefits while minimizing risks.

The exploration of M-EVs in cancer therapy is marked by a commitment to safety and efficacy. Overcoming these challenges will require interdisciplinary collaboration, combining advances in bioengineering, immunology, and clinical research. The refinement of isolation, drug loading, and delivery techniques is pivotal for their successful integration into clinical practice, marking a step forward in personalized and targeted cancer care.

## 8 Conclusion

The exploration of M-EVs represents a pivotal advancement in cancer research, emphasizing their dual role in tumor cell regulation and therapeutic potential. This review highlights how M-EVs can either suppress or promote tumor progression depending on their origin and the tumor microenvironment. While promising, the clinical application of M-EVs faces key challenges, including the need for refined isolation methods, a deeper understanding of their biodistribution and pharmacokinetics, and rigorous safety evaluations. Nonetheless, tumor cell-targeting M-EVs hold immense potential to revolutionize cancer therapy by enabling precise, personalized, and less toxic treatments. With continued innovation, M-EVs are poised to enhance existing therapies and serve as a foundation for developing novel intervention strategies, offering new hope for improved patient outcomes in oncological care.
